# Does an antihypertensive diet cost more? Analysis from the Chinese Heart-Healthy diet trial

**DOI:** 10.1017/S1368980024000430

**Published:** 2024-03-06

**Authors:** Yishan Guo, Danping Su, Hong Chen, Yanxi Ding, Shiyu Zhang, Hong Sun, Dandi Chen, Wenya Yin, Xiang Li, Guo Zeng

**Affiliations:** 1 Department of Nutrition and Food Safety, West China School of Public Health and West China Fourth Hospital, Sichuan University, Chengdu, Sichuan, China; 2 West China School Public Health and West China Fourth Hospital, Sichuan University, Chengdu, Sichuan, China; 3 Research Center for Palliative Care, West China-PUMC C.C. Chen Institute of Health, Sichuan University, Chengdu, Sichuan, China; 4 Sichuan Tourism University, Chengdu, Sichuan, China

**Keywords:** Diet, CHH diet, Food costs, Monetary costs, Hypertension, Sichuan cuisine

## Abstract

**Objective:**

To determine whether the Chinese heart-healthy diet (Sichuan cuisine version) (CHH diet-SC) was more expensive than the conventional Sichuan diet and explore the food groups and nutrients that mainly affected the cost of CHH diet-SC.

**Design:**

Cost analysis of 4-week intervention diets in the Sichuan center representing southwestern China in the CHH diet study.

**Setting:**

A multicentre, parallel-group, single-blind, randomised feeding trial evaluating the efficacy of lowering blood pressure with the cuisine-based CHH diet.

**Participants:**

Totally, fifty-three participants with hypertension aged 25–75 years in the Sichuan center were randomised into the control group (*n* 26) or the CHH diet-SC group (*n* 27).

**Results:**

The CHH diet-SC was more expensive than the control diet (¥27·87 ± 2·41 *v*. ¥25·18 ± 2·79 equals $3·90 ± 0·34 *v*. $3·52 ± 0·39, *P* < 0·001), and the incremental cost-effectiveness ratio for a 1-mm Hg systolic blood pressure reduction was ¥9·12 ($1·28). Intakes and the cost of seafood, dairy products, fruits, soybeans and nuts, whole grains and mixed beans were higher for the CHH diet-SC than for the control diet (*P* < 0·001). Intakes of vitamin B_1_, vitamin B_6_, vitamin C, Mg and phosphorus were positively correlated with the cost (*P* < 0·05).

**Conclusions:**

The CHH diet-SC costs more than the conventional Sichuan diet, partly due to the high cost of specific food groups. Positive correlations between the intakes of vitamin B_1_, vitamin B_6_, vitamin C, Mg, phosphorus and the dietary cost could be a direction to adjust the composition within the food groups to reduce the cost of the CHH diet-SC.

Hypertension is an important risk factor for cardiovascular and kidney diseases, and it has been identified as the leading risk of mortality^(1)^. As a major public health problem, hypertension affects 245 million people in China, with an increasing prevalence rate among adults reaching 27·5 % in 2018^(2)^. The prevalence of hypertension in Sichuan is similar to the national level. Still, the lower rate of control indicates that hypertension as a health issue in the Sichuan region needs more attention^(3)^. For the prevention and treatment of hypertension, the 2017 US Guideline for the Prevention, Detection, Evaluation, and Management of High Blood Pressure in Adults emphasised the importance of nonpharmacological treatment in hypertension management and recommended the Dietary Approaches to Stop Hypertension (DASH) diet^(4)^. However, characteristics including the low consumption of seafood, fruits and dairy products with the heavy oil and salt of the Sichuan diet are far removed from the DASH diet, which partly contributes to the increasing trend of hypertension among Sichuan residents^(5,6)^, so it is essential to change Sichuan cuisine into a balanced and healthy high-quality diet. In 2022, the Chinese heart-healthy (CHH) diet project was optimised to address these shortcomings, and the CHH diet (Sichuan cuisine version) (CHH diet-SC) was introduced to lower blood pressure.

However, whether the CHH diet-SC is more expensive than the conventional Sichuan diet is a question we need to consider. Most cross-sectional studies suggest that high-quality diets such as the DASH diet and Mediterranean diet, which have antihypertensive effects, tend to be more expensive and may pose a barrier to adherence to high-quality diets in the population^(7–9)^. Despite this, a small number of intervention trials have concluded that healthy eating diets may not increase expenditures. Most of these intervention trials are nutrition education trials, providing nutritional guidance for healthy diets like the cardioprotective diet and the Mediterranean diet^(10,11)^. They have improved diet quality to some extent and given participants freedom of food choices, which may explain why the cost of the diets did not increase and still could be afforded by the participants. Thus, the relationship between diets with hypotensive effects and dietary costs remains controversial. In China, there is a lack of studies on the cost of an antihypertensive diet, and only the CHH study briefly evaluated the dietary cost for the first time^(12)^. Our dietary cost analysis of the CHH diet-SC will play an important role in its popularisation in China.

In addition to the analysis of overall dietary consumption, studies have shown that the intakes of specific nutrients in different diets are associated with dietary cost^(13,14)^. High intakes of fibre and minerals such as potassium were found to be potentially correlated with a high dietary cost^(13,15)^. This could provide ideas and a basis for further control of dietary costs. Therefore, the present study aimed to identify whether the CHH diet-SC was more expensive than a conventional Sichuan diet and to explore the food groups and nutrients that primarily affected the cost of the Sichuan diet for further cost reduction.

## Methods

### Study design and participants

The cuisine-based CHH diet project was a multicentre, single-blind, randomised, parallel-controlled feeding trial that compared the effects of the CHH diet with those of the usual local diet (control diet) on reducing blood pressure and other cardiovascular risks among community residents with mild hypertension. The original study methods and further details have been described in the protocol^(16)^. The present study as a complementary study did a secondary data analysis of the CHH study in the Sichuan center by assessing the cost of experimental diets. We recruited fifty-three participants and randomised them to either a CHH diet (*n* 27) or a control diet (*n* 26). The participants were invited to a 7-d run-in phase and then enrolled in the trial for a 4-week intervention. Since our study was a feeding trial, we worked with chefs and dietitians to prepare meals and distribute them to the participants. We chose a canteen as a place for interventions, and participants who had difficulty eating in the canteen could pack their meals to take away. The blood pressure-lowering effects of the CHH diet in the Sichuan center have been analysed previously^(12)^. Experimental diets included three meals a day (breakfast, lunch and dinner) with various dishes, and the menus of meals were developed based on the local dishes and dietary flavour preferences of Sichuan residents.

### Diet development

In our research, we developed experimental diets based on the Dietary Consumption Survey of the Sichuan area to match the characteristics of Sichuan cuisine and the tastes of the participants for good compliance. In addition, we considered the availability and substitutability of foods in different seasons and created a set of unduplicated menus for three meals a day while ensuring food diversity.

We gathered nutritionists, dietitians and chefs to design a 30-day trial dietary menu called the CHH diet-SC for meeting the CHH diet’s daily nutrients and energy composition targets based on the Chinese Dietary Reference Intakes for China (2013)^(17)^, China Food Composition Tables (2019)^(18)^ and The Chinese Dietary Guidelines (2016)^(19)^. The dietary pattern of the CHH diet-SC references the DASH diet rich in fibre, Ca, potassium and protein. Specifically, the CHH-SC reduced energy from fat to 25–27 % with emphasis on the intake of unsaturated fatty acids, raised energy from protein to 17–19 % and energy from carbohydrates to 55–60 %. Considering the essential nutrients that affect hypertension, we raised potassium intake to 3700 mg/d and the intake of fibre to 30 g/d, while reducing Na intake to 2000 mg/d. In addition, low-Na salt and vegetable oil were used in the cooking of the CHH diet-SC.

The control diet was developed according to the national nutrition survey in the Sichuan region, and a slight health adjustment was made to comply with ethics.

### Calculation of the dietary cost

We calculated the whole cost of the 4-week intervention period and the daily dietary cost by using the average daily dietary intake of the participants and the representative retail prices of food in Chengdu city. To accurately assess the food intake of participants, we recorded the precise quantity of food ingredients used in each participant’s meal preparation and weighed the remaining or extra food added after the meals. Due to the complexity of Sichuan cuisine, it was hard to weigh each ingredient in the leftovers, so we only calculated the average weight of each ingredient in the leftovers based on the composition of the original dish. For snacks, we recorded the brand and weight of snacks each participant consumed that were outside the diets in detail.

To come up with a representative cost, we investigated the prices of food consumed in the diets from commonly used sales channels and combined them with food price data released by the Chengdu Municipal Development and Reform Commission (Chengdu.gov.cn). Since the majority of ingredients in the diets are edible agricultural products that are mainly distributed through large-scale wet markets in Chengdu^(20)^, we collected prices of these ingredients through a survey of large-scale wet markets which were also chosen as the source of the official price data released by the Chengdu government. For other pre-packaged seasonings and snacks, we researched the prices in the most common retail supermarket chains in Chengdu, as these products usually have uniform retail prices. All prices were collected during the 4-week intervention period in the trial. In addition to calculating the daily cost, this study assessed the cost and weight of each food group consumed in the two experimental diets. Food was categorised into different food groups according to the China Food Composition Tables (2022)^(21)^ and Chinese Food Guide Pagoda (2022)^(22)^.

### Cost-effectiveness analysis

The dietary cost of the whole 4-week trial was calculated to represent the cost of our study. The outcome of effectiveness was the reduction in systolic blood pressure of the participants in two groups. The diastolic blood pressure was not considered a primary outcome because most of the participants were mildly hypertensive while their diastolic blood pressure was not above the normal range. The primary outcome measure of this study was the incremental cost-effectiveness ratio, calculated as incremental cost (cost of CHH diet-SC–cost of the control group) divided by incremental effectiveness (systolic blood pressure reduction of CHH diet-SC– systolic blood pressure reduction of the control group).

### Assessment of the nutrient intake

We used Nutrition Counter version 2.8.0 to calculate the daily intake of nutrients in two experimental diets that included snacks. The application’s database covered the nutrient content of virtually all food in the diets, with similar substitutes used to fill in the few snack foods that were missing.

### Statistical analysis

All statistical analyses for this study were performed by the statistical software package IBM SPSS Statistics Version 25.0. Independent-sample *t* tests were used to assess the difference between groups for mean costs of daily diets. The differences in amounts of food and the cost of each food group were analysed by using the Wilcoxon rank sum test since the samples did not have a normal distribution. The difference in intake of each nutrient between the two groups was measured by independent-sample t tests or Wilcoxon rank sum tests considering the conditions for the application of statistical methods. Due to the serious multicollinearity among variables, ridge regression was selected in this study to conduct linear regression between daily dietary costs and the intake of nutrients after standardising the energy levels of diets.

## Results

### Baseline characteristics

The baseline characteristics of the two groups are shown in Table 1. A total of fifty-three subjects were randomised into either the control group (*n* 26) or the CHH-SC group (*n* 27).

**Table 1:** Baseline characteristics of participants

Characteristics	CHH diet-SC (*N* 27)	% or SD	Control (*n* 26)	% or SD	All (*n* 53)	% or SD
Gender, *n* (%)						
Male	11	40·7	17	65·4	28	52·8
Female	16	59·3	9	34·6	25	47·2
Age, mean (sd), year	59·77	9·5	60·68	9·3	60·22	9·2
BMI*, mean (sd), kg/m^2^	25·96	2·1	25·93	2·3	25·95	2·1
Education, *n* (%)						
Bachelor’s degree or college and above	2	7·4	5	19·2	7	13·2
High school or technical secondary school	7	25·9	4	15·4	11	20·8
Junior high school or less	18	66·7	17	65·4	35	66·0
Monthly household income, *n* (%)						
>¥ 10 000	9	33·3	7	27·0	16	30·2
¥ 5000–10 000	12	44·4	11	42·3	23	43·4
<¥ 5000	6	22·2	8	30·8	14	26·4
Baseline pressure, mean (sd), mmHg						
SBP^*^	140·42	8·3	140·18	6·7	140·30	7·5
DBP^*^	87·82	8·6	88·29	7·7	88·05	8·1

* SBP, systolic blood pressure; DBP, diastolic blood pressure.

### Differences in dietary cost between the two diets

Comparing the daily dietary cost based on the food prices in the current season between the two diets, we found that the CHH diet-SC was significantly more expensive than the control diet. (*P* < 0·001; Table 2). After subtracting the cost of snacks, the mean difference in daily dietary cost between the two groups rose from ¥2·69 ($0·38), accounting for 13·4 %, to ¥3·44 ($0·48), accounting for 18·2 %. This indicates that the gap in the costs between the CHH diet-SC and the control diet was greater in the original study design. In addition, we evaluated the cost-effectiveness of the CHH diet-SC *v*. the control diet at the end of the trial, and the incremental cost-effectiveness ratio for a 1-mmHg systolic blood pressure reduction was ¥9·12 ($1·28) (Table 3).

**Table 2:** Daily cost and mean difference in cost of diet for CHH diet-SC and control diets^*^

	CHH diet-SC (*n* 28) (¥/d)		Control (*n* 28) (¥/d)		Mean difference (¥/d)	95 % CI	*P* value
	Mean	sd	Mean	sd			
With snacks	27·87	2·41	25·18	2·79	2·69	1·83, 3·55	<0·001
Without snacks	27·21	2·43	23·76	2·77	3·44	2·57, 4·31	<0·001

* USD to CNY exchange rate in December 2023: $1= ¥7.15.

**Table 3:** Incremental cost-effectiveness ratio for SBP reduction

Interventions	Cost*, ¥	Incremental cost, ¥	Effects^†^, mean	95 % CI	Incremental effect^‡^, mean	95 % CI	ICER, ¥/ mmHg
CHH diet-SC	21 069·72	2738·63	−15·43	–18·48, –12·37	−10·95	–16·09, –5·82	9·12
The control diet	18 331·04	N/A^†^	−4·47	–8·36, –0·60	N/A^†^		

SBP, systolic blood pressure.

* USD to CNY exchange rate in December 2023: $1= ¥7.15.

† Effects refers to the SBP reduction.

‡ N/A: not applicable.

The differences in the daily consumption and costs of food groups between the two diets can be seen in Table 4. The amount and cost of dairy products, fruits, soybeans and nuts were higher for the CHH diet-SC than for the control diet, while edible oil, salt and snacks presented the opposite results. Notably, there was no significant difference in the average daily consumption of cereals or tubers between the two groups (*P* = 0·678), but the cost of cereals and tubers in the CHH diet-SC was more expensive than in the control diet (*P* < 0·001), probably due to the higher cost caused by the larger daily consumption of whole grains and beans in the CHH diet-SC. Similarly, there was no significant difference between the two diets in average daily consumption or cost of vegetable and animal food. However, the composition of animal food was different, with the proportion of seafood and eggs being higher in the CHH diet-SC than in the control diet, while the proportion of meat was lower.

**Table 4:** Daily amount and cost of food groups in CHH diet-SC and control diets^*^

Food group	CHH diet-SC (*n* 70)		Control (*n* 70)		*P* value
	Mean	sd	Mean	sd	
Cereals and tubers					
g/d	382·19	65·15	386·84	65·06	0·678
¥/d	2·99	0·62	2·33	0·41	<0·001
Whole grains and mixed beans					
g/d	152·93	61·83	37·57	47·77	<0·001
¥/d	1·58	0·62	0·30	0·37	<0·001
Refined grains					
g/d	212·10	32·31	306·49	31·48	<0·001
¥/d	1·33	0·24	1·83	0·21	<0·001
Tubers					
g/d	17·16	42·19	42·78	42·84	<0·001
¥/d	0·07	0·17	0·20	0·22	<0·001
Vegetables					
g/d	433·15	76·10	445·17	89·61	0·394
¥/d	3·19	1·09	3·19	0·82	0·978
Animal food					
g/d	205·86	34·09	198·84	26·96	0·179
¥/d	11·45	2·13	11·46	2·26	0·997
Meat					
g/d	133·59	24·31	162·04	28·32	<0·001
¥/d	9·21	2·58	10·86	2·30	<0·001
Eggs					
g/d	42·19	29·56	27·52	27·34	0·010
¥/d	0·50	0·37	0·33	0·33	0·013
Seafood					
g/d	30·08	33·77	9·28	23·39	<0·001
¥/d	1·75	1·58	0·28	0·70	<0·001
Dairy products					
g/d	260·92	37·72	79·30	125·52	<0·001
¥/d	3·26	0·47	0·83	1·34	<0·001
Fruits					
g/d	231·60	85·18	51·05	69·55	<0·001
¥/d	2·14	0·76	0·47	0·66	<0·001
Soybeans and nuts					
g/d	34·80	19·31	17·95	19·26	<0·001
¥/d	0·93	0·80	0·94	1·29	<0·001
Cooking oil					
g/d	25·42	2·43	42·39	6·15	<0·001
¥/d	2·67	0·37	3·84	0·94	<0·001
Salt					
g/d	4·00	1·14	7·22	1·42	<0·001
¥/d	0·05	0·01	0·06	0·01	<0·001
Snacks					
g/d	57·07	42·05	82·73	34·83	<0·001
¥/d	0·66	0·50	1·41	0·79	<0·001

* USD to CNY exchange rate in December 2023: $1= ¥7.15.

### Regression analysis of the intake of nutrients and dietary cost of the CHH diet-SC

The differences in the nutrient intake of the two diets are presented in Table 5. The CHH diet-SC significantly raised the percentage of energy from protein and carbohydrates while reducing that from fat. In addition, the CHH diet-SC contained more macronutrients and minerals, especially potassium, Ca, Fe, fibre, vitamin C and B vitamins, which are beneficial for lowering blood pressure. We reduced the Na intake in the CHH-SC to < 3 g as much as possible.

**Table 5:** Daily intake of nutrients in the CHH diet-SC and control diets

Nutrients	CHH diet-SC (*n* 70)		Control (*n* 70)		*P* value
	Mean	sd	Mean	sd	
Energy (kcal/d)	2252·97	145·46	2400·24	144·12	<0·001
Carbohydrate (%E)	57·37	2·47	52·74	3·67	<0·001
Protein (%E)	17·90	1·15	12·84	1·70	<0·001
Fat (%E)	24·80	2·11	34·44	3·86	<0·001
Cholesterol (mg/d)	412·19	155·57	340·16	139·83	0·026
Fibre (g/d)	29·52	3·48	13·80	5·30	<0·001
Vitamin A (μgRAE/d)	1042·83	946·39	668·14	964·18	<0·001
Vitamin D (μg/d)	12·25	7·82	6·55	12·85	<0·001
Vitamin E (mg/d)	46·94	7·13	40·68	8·92	<0·001
Vitamin B_1_ (mg/d)	1·31	0·15	1·13	0·13	<0·001
Vitamin B_2_ (mg/d)	1·37	0·28	0·86	0·38	<0·001
Vitamin B_6_ (mg/d)	0·56	0·12	0·47	0·17	<0·001
Vitamin C (mg/d)	152·22	36·19	120·75	56·96	<0·001
Folic acid (μg/d)	266·27	124·47	184·14	80·58	<0·001
Ca (mg/d)	980·34	98·81	420·86	131·27	<0·001
Phosphorus (mg/d)	1687·89	126·05	1128·89	430·56	<0·001
Potassium (mg/d)	4108·74	384·44	2357·30	850·26	<0·001
Na (mg/d)	2492·52	343·29	5898·47	3245·76	<0·001
Mg (mg/d)	550·39	66·95	316·17	72·33	<0·001
Se (μg/d)	47·61	12·61	36·36	7·47	<0·001
Cu (mg/d)	3·85	1·84	1·90	1·45	<0·001

To explore the intake of which nutrients would affect the cost of the CHH diet-SC, we first used single-factor analysis to analyse the relationship between each nutrient and daily dietary cost. We found that energy intake affected the dietary cost in this study, which was beyond our expectations because the daily energy intake of the CHH diet-SC should not have fluctuated greatly according to the original study design. To eliminate the impact, we performed a linear regression of dietary nutrients and cost after standardising energy intake. Ridge regression was used to establish the model, and the variation in daily dietary cost was mostly attributed to vitamin B_1_, vitamin B_6_, vitamin C, Mg, phosphorus and Se (Table 6).

**Table 6.** Ridge regression analysis between intakes of nutrients and dietary cost^*^

Nutrients (mg/d)	Standardised coefficients (*β*)	*P* value
Vitamin B_1_	0·21	0·003
Vitamin B_6_	0·14	0·020
Vitamin C	0·12	0·033
Mg	0·24	0·009
Phosphorus	0·28	0·003
Se	0·15	0·022

* Model built after standardising the intakes of energy.

## Discussion

The key finding of this study is that the CHH diet-SC designed to reduce cardiovascular risk factors such as blood pressure was more expensive than the control diet representative of the eating habits in the Sichuan region (¥27·87 ± 2·41 *v*. ¥25·18 ± 2·79 equals to $3·90 ± 0·34 *v*. $3·52 ± 0·39). This is mainly because increasing the intake of certain food groups, such as fruits, whole grains and mixed beans and dairy products, substantially results in higher costs for these food groups. Using the total costs for the two groups in the trial and the reduction in systolic blood pressure of the participants, we evaluated the incremental cost-effectiveness ratio for systolic blood pressure reduction as ¥9·12 ($1·28) per mmHg, indicating a superior economic benefit of the CHH diet-SC compared with the main study ($1·62 per mmHg)^(12)^. Exploring the relationship between the intakes of nutrients and the cost of the Sichuan diet to provide a reference for lowering dietary cost, vitamin B_1_, vitamin B_6_, vitamin C, Mg, phosphorus and Se were found to principally affect the dietary cost.

The present study is the first to specifically analyse the cost of an antihypertensive diet in China, which enriches the related research evidence. Moreover, there is no consensus to date on whether diets with blood-pressure-lowering effects are more expensive. Our findings are consistent with the findings of most observational studies and an LSHP intervention study, showing that antihypertensive diets cost more^(15,23–25)^. Although the CHH diet-SC was found to be more expensive in our study, its cost was much lower than the cost of a healthy diet in eastern Asia at $4·72 in 2020, which was estimated in ‘The State of Food Security and Nutrition in the World 2022’^(26)^. There are few studies assessing the dietary cost in China, with one study calculating the cost of the diet recommended by the Chinese Dietary Guideline (2016). Since it used the prices of food in rural Chengdu from the Sichuan Statistical Yearbook, the cost of the diet was only ¥14·44 ($2·02)^(27)^, which is much lower than the cost of the CHH diet-SC. Data released by the National Bureau of Statistics of China indicated that per capita food consumption expenditure (including tobacco and alcohol) in 2020 was ¥17·48 ($2·44)^(28)^. It may seem like the cost of CHH diet-SC is a little expensive in China, but it is important to consider that Chengdu, as the capital city of Sichuan Province, has become a new first-tier city in the country since 2013. Its economic competitive power is among the highest in China, so it is rational that both of the two experimental diets are more expensive than per capita food consumption expenditure (including tobacco and alcohol) of the whole country. In addition, observational studies using various dietary quality evaluation indices to explore the correlation between dietary cost and the quality of the diet also concluded that a healthier diet requires higher cost^(29–31)^. This is mainly because the high cost of a healthy diet may be due to ample healthy foods (nutrient-rich foods such as fruits and vegetables), which tend to cost more per calorie than energy-dense foods such as candy and grains according to a previous study^(32)^. In our study, the costs of nutrient-rich foods such as whole grains and mixed beans, dairy products (mainly low-fat milk), soybeans and nuts, fruits, seafood and eggs, as recommended by the Chinese Dietary Guideline and DASH diet, were higher in the CHH diet-SC group than in the control group, which made the CHH diet-SC more expensive. Similarly, studies from the UK and Malaysia explored that the dietary cost was higher when the consumption of fruits, soybeans and nuts, as well as low-fat dairy products, was closer to the requirements of a healthy diet^(8,29)^. In addition, researchers have found that the consumption of these nutrient-rich food groups showed a positive correlation with people’s socio-economic position, which also suggested that increasing the intake of these food groups may be unaffordable for some people^(33)^.

However, it was reported that nutritious and cheaper foods within those food groups do exist, but they are usually not chosen or accepted by the majority of the population because of their taste, cultural preferences, etc^(32)^. Therefore, reducing the cost of specific food groups without compromising the quality of diet is a priority. Setting up subsidies should be considered since many countries have subsidised the purchase of nutritious food such as vegetables and fruits and were proven to obtain good health value^(33–36)^. Thus, subsidisation of healthy diets should be part of the overall action plan for hypertension prevention and control.

In contrast, the result that the CHH diet-SC cost more in our study is not consistent with the body of evidence from trials providing nutrition education interventions^(10,11,37)^. Some factors may explain the conflicting results. The diets developed in our trial were based on the dietary habits of the residents in the Sichuan province of China, which had a huge difference from the diets derived from dietary habits in Western countries from other trials. This can lead to differences in the gap between the diet at baseline and a healthy diet since the compositions of food groups in various healthy dietary patterns have many similarities. According to the statistical report of Sichuan Province in 2020, the average daily consumption of fruits, dairy products and seafood by Sichuan residents was only 40·77 g, 10·04 g and 9·21 g, respectively^(38)^. The low intakes of fruits, dairy products and seafood in the control group in our study reflected these defects in the Sichuan diet. However, the nutrition education study from the UK, which explored the cost of a cardioprotective diet, showed that the participants consumed 211·2 g, 250·0 g and 60·3 g of fruits, dairy products and seafood at a high level in their diets at baseline^(10)^. This led to no change in their intake of food groups after the interventions, and the total dietary cost did not appear to be more expensive in the end, which was inconsistent with the conclusions of our study. In addition, although the interventions for nutrition education in most trials have a certain biological effect, observing the growth of various healthy dietary indices in different trials showed that they had limitations on the improvement of participants’ diets^(10,11,37)^. Since the present study was a feeding trial, we strictly controlled the intake of food in each meal to follow the design criteria, and the subject’s compliance appeared to be good, which improved the diet quality more substantially than the behavioral intervention trials. This may result in higher costs, leading to different conclusions in the cost analysis of the behavioural intervention trials and the feeding trials.

Compared with the conventional diet in Sichuan, the CHH diet-SC is rich in protein, fibre, various vitamins and minerals. Our study found that increasing the intake of vitamin B_1_, vitamin B_6_, vitamin C, Mg, phosphorus and Se, which affected blood pressure^(38–43)^, was associated with a greater cost of the CHH diet-SC. This is mainly because the main sources of these nutrients are whole grains and mixed beans, soybeans and nuts, dairy products, seafood and fruits, of which the intake and monetary cost have a significant difference between the CHH diet-SC and the control diet. However, the correlation we explored was only partially consistent with the findings of other studies. In addition to the nutrients we found to be positively correlated with dietary cost, studies from the United States and Japan have found similar associations between cost and other nutrients, such as potassium, Fe and fibre, which were also beneficial for lowering blood pressure^(13,14,41)^. Of note, multiple studies have shown that potassium-rich diets tend to cost more^(13–15)^, while potassium intake was not a factor affecting the cost of the Sichuan diet in our study. This may be because the low Na salt containing high potassium chosen by the CHH diet-SC has become a cost-effective food source of potassium except for fruits and vegetables.

In summary, the findings of studies exploring the nutrients related to dietary cost are not exactly the same because the main food sources of nutrients consumed by people in different regions are varied, and the prices of the same food are also different due to taxes, production, and other reasons. This study aimed to provide a basis for lowering the cost of the CHH diet-SC by identifying the nutrients that had the main effects. Drawing on some studies that have explored the most cost-effective food sources of various nutrients^(44,45)^, we can improve the menus of the CHH diet-SC by choosing more cost-effective food sources of vitamin B_1_, vitamin B_6_, vitamin C, Mg, phosphorus and Se to narrow the cost gap between the CHH diet-SC and the conventional diet in Sichuan while maintaining the current intake of each nutrient and food group.

The strengths of our study include the use of a randomised clinical feeding trial to specifically assess the cost of an antihypertensive diet in China for the first time. The actual food intake of the participants was accurately recorded, and the food prices were collected through the national government report and market surveys, which made the data on the dietary cost more reliable. In addition, we explored the main nutrients that affected the dietary cost of the Sichuan diet, providing directions and a basis for reducing the cost in the future. However, some limitations should be considered. Compared with educational intervention trials and observational studies, the affordability of the CHH diet-SC needs to be further confirmed in the population. The food prices we collected only represented Chengdu city, so extrapolating the conclusion to other areas of Sichuan should be performed with caution.

In conclusion, the present study showed that the CHH-SC diet which reduced blood pressure was more expensive than the conventional Sichuan diet. The high cost of specific healthy food groups in the CHH diet-SC could be the main reason for the expensive dietary cost. Thus, subsidies for these food groups should be considered to reduce prices and promote purchases. Furthermore, the exploration of the positive correlation between vitamin B_1_, vitamin B_6_, vitamin C, Mg, phosphorus, Se and the cost of the Sichuan diet suggests that we can reduce the cost of the CHH diet-SC by selecting cost-effective food sources of these nutrients within food groups in the future.
